# Purifying and profiling lysosomes to expand understanding of lysosomal dysfunction–associated diseases

**DOI:** 10.1172/JCI188507

**Published:** 2025-02-17

**Authors:** Ali Shilatifard, Issam Ben-Sahra

**Affiliations:** Northwestern University, Feinberg School of Medicine, Chicago, Illinois, USA.

## Abstract

Lysosome storage dysfunction plays a central role in numerous human diseases, but a lack of appropriate tools has hindered lysosomal content profiling in clinical settings. In this issue of the *JCI*, Saarela et al. introduce a method called tagless LysoIP that enabled rapid isolation of intact lysosomes from blood and brain cells via immunoprecipitation of the endogenous protein TMEM192. Applied to the neurodegenerative lysosomal storage disorder known as Batten disease (caused by mutations in the *CLN3* gene), tagless LysoIP revealed substantial accumulation of glycerophosphodiesters (GPDs) in patient lysosomes. These findings highlight the role of CLN3 in GPD clearance and present an innovative method that will enable biomarker discovery and therapeutic advancement in lysosomal diseases.

## Central role for lysosomes in signaling and disease

Historically regarded as cellular “recycling bins,” lysosomes have emerged as dynamic organelles integral to cellular homeostasis (1). Beyond degrading cellular debris, lysosomes regulate key metabolic and signaling pathways, including nutrient sensing via the mechanistic target of rapamycin complex 1 (mTORC1) (2). These activities position lysosomes as pivotal hubs at the intersection of metabolism, signaling, and disease (3, 4). In lysosomal storage disorders, loss-of-function mutations in lysosomal enzymes or transporters lead to the accumulation of undegraded substrates, causing progressive cellular and organ damage (5). These rare genetic conditions can also affect the central nervous system where they lead to progressive neurocognitive impairment and seizures, such as seen in Tay-Sachs and Niemann-Pick disease. The broad range of potential cell types affected by lysosomal dysfunction emphasizes the centrality of lysosomes in normal cellular function (6). However, lysosomal dysfunction is not limited to rare diseases. It is also increasingly implicated in neurodegenerative conditions like Alzheimer’s disease (AD) and Parkinson’s disease (PD), in which defective protein degradation contributes to the accumulation of toxic aggregates (7). Because lysosomal dysfunction can affect many different proteins, profiling lysosomal content from clinical samples can inform specific disease mechanisms. In this issue of the *JCI*, Saarela and colleagues (8) describe the development of tagless LysoIP, a method that enables the isolation and analysis of lysosomes directly from patient-derived cells, marking a substantial advance in lysosomal research.

## The tagless LysoIP method for clinical lysosome profiling

**Figure 1:**
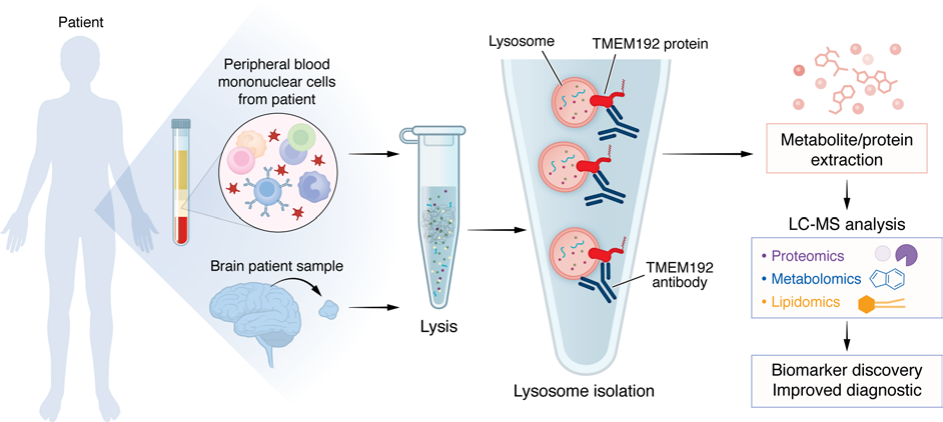
The tagless LysoIP method isolates lysosomes and supports biomarker discovery from clinical samples. The workflow for tagless LysoIP starts with the collection of samples such as PBMCs or tissues from patients. Cells are lysed to release intracellular components, and lysosomes are specifically isolated using an antibody targeting the endogenous lysosomal protein TMEM192. This method ensures the rapid and selective capture of intact lysosomes without the need for genetic tagging. Isolated lysosomes undergo metabolite and protein extraction, enabling comprehensive profiling through liquid chromatography-mass spectrometry (LC-MS). This workflow supports proteomics, as well as application to metabolomics and lipidomics analyses, to facilitate discovery of lysosome-specific biomarkers, advance our understanding of lysosomal dysfunction, and improve diagnostic and therapeutic strategies for lysosome-driven diseases.

By targeting TMEM192, an endogenous lysosomal transmembrane protein, the tagless LysoIP method isolated intact lysosomes from patient-derived samples, such as peripheral blood mononuclear cells (PBMCs) or induced pluripotent stem cell–derived (iPSC-derived) neurons, without the need for genetic manipulations. This feature sets the tagless LysoIP apart from traditional tagged methods,such as LysotagIP (9), that require lentiviral transduction or other methods to introduce a tag. Tagless LysoIP enables access to lysosome-specific biochemical analyses, including lysosomal proteomics, metabolomics, and lipidomics (Figure 1). This capability unlocks exciting opportunities for biomarker discovery and the elucidation of disease mechanisms. Tagless LysoIP holds particular promise for clinical translational research; it provides a practical, minimally invasive tool for identifying lysosome-related biomarkers, which can be used to track disease progression and assess therapeutic responses. Without the need for genetic tagging, this method is broadly applicable to real-world clinical samples, allowing researchers to capture lysosomal anomalies and biochemical profiles that could serve as critical markers in lysosomal storage disorders, neurodegenerative diseases, and other conditions where lysosomal dysfunction plays a pivotal role.

## Glycerophosphodiesters accumulate in Batten disease

To validate the clinical utility of their method, Saarela et al. (8) applied tagless LysoIP to patients with Batten disease, an autosomal recessive neurodegenerative disorder characterized by progressive neuronal ceroid lipofuscinosis. Batten disease typically manifests in childhood and is marked by progressive neurological decline due to mutations in the *CLN3* gene, which encodes a transmembrane lysosomal/endosomal protein needed for normal lysosomal function. Recent studies have suggested a role for CLN3 in glycerophosphodiester (GPD) metabolism within the lysosome (10, 11). Using tagless LysoIP, Saarela et al. (8) isolated lysosomes from PBMCs of patients with Batten disease and found elevated levels of GPDs within these lysosomes. GPDs such as glycerophosphoinositol (GPI), glycerophosphoglycerol (GPG), and glycerophosphocholine (GPC) were markedly more abundant in lysosomes from Batten PBMCs than in those from healthy controls. This finding provides direct evidence of lysosomal GPD accumulation in human Batten disease, echoing findings in preclinical models (10) and highlighting CLN3’s role in GPD clearance. Importantly, GPD accumulation was detected specifically within lysosomal isolates, emphasizing the necessity of lysosome-specific enrichment to detect these pathogenic changes.

Interestingly, a patient with a milder Batten phenotype exhibited lower levels of lysosomal GPD accumulation, suggesting that GPD levels might correlate with disease severity. This finding positions GPD levels as a potential biomarker for monitoring disease progression or therapeutic efficacy, opening avenues for personalized approaches to treat people with Batten disease.

## Clinical implications and broader applications

The clinical implications of the tagless LysoIP method are far reaching. Traditional assays for lysosomal disorders focus on measuring enzyme deficiencies or substrate accumulation (12) but lack the depth and specificity provided by direct lysosomal content profiling. By enabling the isolation and analysis of lysosomal content from patient samples, tagless LysoIP could transform how lysosomal storage disorders are diagnosed, monitored, and treated.

The tagless LysoIP method could be extended to other neurodegenerative disorders involving lysosomal or autophagic impairments, such as PD and AD. Lysosomal dysfunction is implicated in PD through the impaired clearance of α-synuclein aggregates (13, 14). Similarly, AD research increasingly links autophagy-lysosome pathways with the accumulation of amyloid and tau proteins, which disrupt cellular homeostasis (15, 16). Although brain samples are not readily available from living patients with AD or PD, this method can be applied to surrogate cell types that maybe more readily accessible via blood, skin, and urine. Patient cells can also be used to generate induced pluripotent stem cells (IPSCs) that can differentiated into surrogate cell types useful for lysosome profiling, although the need to make IPSCs limits real-time assessment. Profiling lysosomal content from patient-derived cells could not only deepen our understanding of these complex neurodegenerative conditions but also uncover new lysosomal biomarkers for disease progression or the efficacy of treatments targeting lysosomal pathways. Lysosomal function also declines with age, and this decline is thought to contribute to various age-associated diseases, including certain cancers and metabolic disorders (17–19). By profiling lysosomal content across the lifespan age-related changes in lysosomal function can be monitored, enabling potential interventions aimed at restoring normal lysosomal function to support healthy aging. The tagless LysoIP method could therefore be especially transformative in geriatrics, where lysosomal function could serve as an indicator of cellular health.

## A platform for biomarker-guided therapies and drug discovery

Beyond diagnostics, the tagless LysoIP presents a promising platform for biomarker-guided therapy in lysosomal disorders. In Batten disease, where lysosomal GPD accumulation reflects disease activity, GPD levels could help evaluate therapeutic efficacy. For example, a reduction in GPD levels following treatment could signal a positive response, allowing for timely adjustments in therapeutic strategies. It might also be applied to monitor enzyme replacement therapy in Niemann Pick disease. In this setting, the source material obtained from the patient needs to reflect the target treatment tissue in order provide an assessment of treatment efficacy. This approach could also extend to other lysosomal storage disorders where substrate accumulation within lysosomes acts as a measurable marker of disease progression and therapeutic impact, particularly relevant in treatments such as enzyme replacement therapies or pharmacological chaperones. However, to maximize applicability, further research is needed to understand the stability of these lysosomal biomarkers and how they may vary in response to therapy or disease progression. Understanding these aspects will be crucial to ensure that these biomarkers provide reliable, accurate information about treatment efficacy. The tagless LysoIP method also has substantial potential for drug discovery, particularly by enabling high-throughput screening of compounds aimed at modulating lysosomal function in a human-relevant context. Using lysosomes directly isolated from patient cells, researchers can gain detailed insights into a compound’s efficacy and safety profile, specifically within lysosomal pathways. By bridging the gap between basic lysosomal research and translational applications, tagless LysoIP could accelerate the discovery and development of therapies for a range of lysosomal disorders.

## Conclusion and future directions

The tagless LysoIP method introduced by Saarela et al. (8) represents a transformative advance in lysosomal research, bridging the gap between basic science and clinical application. By applying this technique to Batten disease, the authors demonstrate how it can be used to uncover lysosome-specific changes in human disorders, advancing our ability to identify biomarkers and develop targeted lysosomal therapies. Looking ahead, this method has immense potential in translational research, with applications spanning from age-related diseases to complex neurodegenerative disorders. However, the method’s scalability remains a challenge for widespread clinical application. For tagless LysoIP to transition from research to routine use, it would need to be refined for high-throughput and cost-effective processing, ensuring consistency across various laboratories. Additionally, the technical requirements and antibody dependence of the method may limit its feasibility in settings lacking advanced laboratory infrastructure or resources and this is highly relevant for clinical laboratories. Addressing these scalability concerns will be essential for realizing the method’s full potential. For researchers, the tagless LysoIP nonetheless provides a practical, minimally invasive way to evaluate lysosomal health, opening exciting possibilities for new diagnostics and treatments in lysosomal storage disorders and beyond. As our understanding of lysosomal biology extends across various diseases, the capacity to study lysosomes directly from patient samples is set to substantially impact clinical practice and broaden our understanding of lysosomal disease mechanisms.

Address Correspondence to: Ali Shilatifard, 303 E. Superior Street, 7-516, Chicago, Illinois, 60611, USA. Phone: 312.503.5217; Email: ASH@northwestern.edu. Or to: Issam Ben-Sahra 303 E. Superior Street, 7-520, Chicago, Illinois, 60611, USA. Phone: 312.503.5318; Email: issam.ben-sahra@northwestern.edu.

## Version 1 02/17/2025

Electronic publication
